# Medicated Scaffolds Prepared with Hydroxyapatite/Streptomycin Nanoparticles Encapsulated into Polylactide Microfibers

**DOI:** 10.3390/ijms23031282

**Published:** 2022-01-24

**Authors:** Amirmajid Kadkhodaie-Elyaderani, Maria del Carmen de Lama-Odría, Manuel Rivas, Immaculada Martínez-Rovira, Ibraheem Yousef, Jordi Puiggalí, Luis J. del Valle

**Affiliations:** 1 Departament d’Enginyeria Química, Universitat Politècnica de Catalunya, EEBE, Av. Eduard Maristany 10-14, E-08019 Barcelona, Spain; amirmajid.kadkhodaie@upc.edu (A.K.-E.); mcdlo1998@gmail.com (M.d.C.d.L.-O.); manuel.rivas-canas@upc.edu (M.R.); 2 Barcelona Research Center in Multiscale Science and Engineering, Universitat Politècnica de Catalunya, Campus Diagonal-Besòs, Av. Eduard Maristany 10-14, E-08019 Barcelona, Spain; 3 MIRAS Beamline BL01, ALBA-CELLS Synchrotron, Carrer de la Llum 2-26, E-08290 Cerdanyola del Vallès, Barcelona, Spain; immamartinez@gmail.com (I.M.-R.); iyousef@cells.es (I.Y.); 4 Ionizing Radiation Research Group, Physics Department, Universitat Autònoma de Barcelona (UAB), E-08193 Cerdanyola del Vallès, Barcelona, Spain; 5 Institute for Bioengineering of Catalonia (IBEC), The Barcelona Institute of Science and Technology (BIST), Carrer Baldiri i Reixac 11-15, E-08028 Barcelona, Spain

**Keywords:** hydroxyapatite nanoparticles, streptomycin, polylactide, drug delivery, antimicrobial activity, cytotoxicity

## Abstract

The preparation, characterization, and controlled release of hydroxyapatite (HAp) nanoparticles loaded with streptomycin (STR) was studied. These nanoparticles are highly appropriate for the treatment of bacterial infections and are also promising for the treatment of cancer cells. The analyses involved scanning electron microscopy, dynamic light scattering (DLS) and Z-potential measurements, as well as infrared spectroscopy and X-ray diffraction. Both amorphous (ACP) and crystalline (cHAp) hydroxyapatite nanoparticles were considered since they differ in their release behavior (faster and slower for amorphous and crystalline particles, respectively). The encapsulated nanoparticles were finally incorporated into biodegradable and biocompatible polylactide (PLA) scaffolds. The STR load was carried out following different pathways during the synthesis/precipitation of the nanoparticles (i.e., nucleation steps) and also by simple adsorption once the nanoparticles were formed. The loaded nanoparticles were biocompatible according to the study of the cytotoxicity of extracts using different cell lines. FTIR microspectroscopy was also employed to evaluate the cytotoxic effect on cancer cell lines of nanoparticles internalized by endocytosis. The results were promising when amorphous nanoparticles were employed. The nanoparticles loaded with STR increased their size and changed their superficial negative charge to positive. The nanoparticles’ crystallinity decreased, with the consequence that their crystal sizes reduced, when STR was incorporated into their structure. STR maintained its antibacterial activity, although it was reduced during the adsorption into the nanoparticles formed. The STR release was faster from the amorphous ACP nanoparticles and slower from the crystalline cHAp nanoparticles. However, in both cases, the STR release was slower when incorporated in calcium and phosphate during the synthesis. The biocompatibility of these nanoparticles was assayed by two approximations. When extracts from the nanoparticles were evaluated in cultures of cell lines, no cytotoxic damage was observed at concentrations of less than 10 mg/mL. This demonstrated their biocompatibility. Another experiment using FTIR microspectroscopy evaluated the cytotoxic effect of nanoparticles internalized by endocytosis in cancer cells. The results demonstrated slight damage to the biomacromolecules when the cells were treated with ACP nanoparticles. Both ACP and cHAp nanoparticles were efficiently encapsulated in PLA electrospun matrices, providing functionality and bioactive properties.

## 1. Introduction

Hydroxyapatite (HAp) (Ca_10_(PO_4_)_6_(OH)_2_) is probably the most important type of calcium phosphate ceramic due to its increasing application in biomedicine. HAp features a relative resemblance to the inorganic mineral component of bone and teeth; consequently, devices based on HAp can possess special biocompatibility and unique bioactivity. Calcium phosphates can be found in different individual phases (e.g., amorphous calcium phosphate (ACP), tricalcium phosphate (TCP), tetracalcium phosphate (TTCP), octacalcium phosphate (OCP) and hydroxyapatite (HAp)), which vary in composition, Ca/P ratio (e.g., calcium-deficient HAp), crystalline structure, degree of substitution (e.g., carbonate substituted HAp) and properties (e.g., different solubility and bioresorbability) [1,2,3,4,5,6]. Although none of these phases can render materials with ideal properties, great efforts are focused on the development of multiphasic systems based on calcium phosphates.

The porosity of HAp offers a significant opportunity for the encapsulation of pharmacological substances, such as antibody fragments, antibiotics, hormones and enzymes. This potential makes HAp a suitable biomaterial to deliver pharmacological substances with sustained release capacity in the treatment of bone problems and cancers in which local delivery helps to fill defects in the skeleton. Due to these excellent roles of HAp in biomedical applications [7], several methods have been considered for the preparation or synthesis of HAp nanoparticles with optimized and close properties to living hard tissues, such as bones and teeth. These methods include, among other techniques, chemical precipitation [8,9,10], sol-gel [8], hydrothermal [11], multiple emulsion [12], biomimetic deposition [13,14] and electrodeposition [15].

Conventional drug administration (e.g., tablets or intravenous injections) features disadvantages, such as the problems caused by the delivery of a complete dose in a short period of time (e.g., toxicity and unpleasant side effects). The development of controlled drug delivery systems has become essential to reduce drug concentrations and provide long-term therapeutic effects [16,17,18,19]. HAp has been applied substantially as a carrier for the controlled delivery of different types of therapeutic agent (e.g., drugs, genes, antigens, enzymes, proteins) [20,21]. Basic factors in drug release, such as pore size, surface area and surface functionalization can be easily controlled in HAp systems [22]. For example, Xu et al. [23] demonstrated that coating HAp particles with hydrophobic liposomes could decrease the release rate of hydrophobic drugs (i.e., 20 h were necessary to deliver 70% of encapsulated indomethacin, while only 5 h were required for uncoated samples).

**Figure 1:**
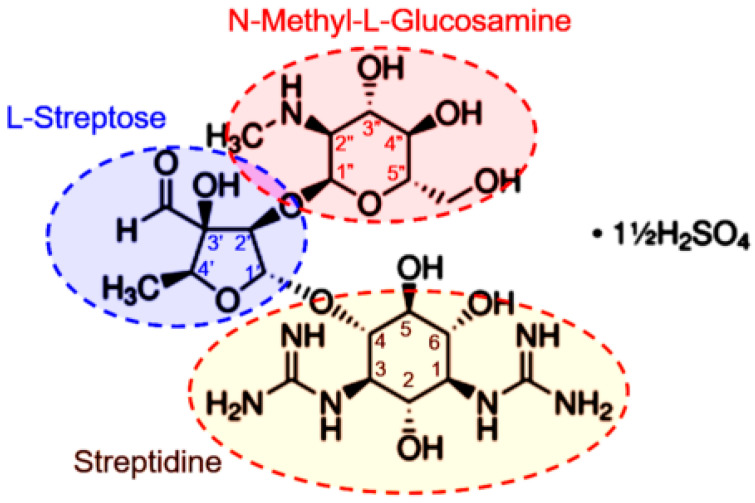
Chemical structure of STR sulfate.

Streptomycin (STR) is the oldest broad-spectrum antibiotic (i.e., known since 1940) used in the treatment of bacterial infections, such as tuberculosis caused by *Mycobacterium tuberculosis* [24,25]. Streptomycin is a prototype of aminoglycoside antibiotics (Figure 1), which essentially acts by binding to the 16S rRNA of the 30S subunit of the bacterial ribosome, thereby preventing protein synthesis through a complex pathway [26]. STR was widely used until the widespread emergence of bacterial resistance limited its clinical usefulness. The mechanism of microbial resistance to streptomycin has been reviewed extensively elsewhere [27]. Several efforts have been made to restore the usefulness of streptomycin and other aminoglycosides. These include chemical modifications to develop new generations of aminoglycosides that bypass existing multidrug bacterial resistance pathways and reduce concerns about their toxicity, especially nephrotoxicity [28]. Despite their limitations, including bacterial resistance, poor oral pharmacokinetics, clinical toxicity and side effects, aminoglycosides (including the prototype streptomycin) remain clinically significant antimicrobial agents in the treatment of a broad range of bacterial infections [28,29]. In addition, STR is currently the subject of major interest as an effective drug against cancer due to its potential capacity to interfere with mitochondrial biogenesis [30,31]. Mitochondria feature a prokaryote origin, as do bacteria; consequently, they can be affected by STR. This point is fundamental, since cancer cells require very high mitochondrial activity to survive and reproduce.

The encapsulation of STR is also important in order to prevent drugs from being expelled from the interior of cells through molecular pumps. HAp is obviously an ideal encapsulation agent for STR due to its biocompatibility and its capacity to be transfected via endocytosis into cells. The slow release of STR from particles seems necessary in order to avoid ejection via the activation of cell ejection pumps [27]. Finally, it also seems interesting to produce a biodegradable polymeric matrix that could act as a reservoir of STR-loaded HAp particles.

The goals of the present work can be summarized by the following steps: (a) Encapsulation of STR in HAp nanoparticles with different physicochemical characteristics (e.g., degree of crystallinity); (b) evaluation of the STR release from nanoparticles loaded by different methods; (c) preparation of Hap-loaded, electrospun scaffolds based on polylactide as an example of a biodegradable polymer; (d) evaluation of antibacterial properties of Hap-loaded systems as well as the effect of loaded fibers and nanoparticles on tumoral cells.

## 2. Results and Discussion

### 2.1. Characterization of Hydroxyapatite Particles

**Figure 2:**
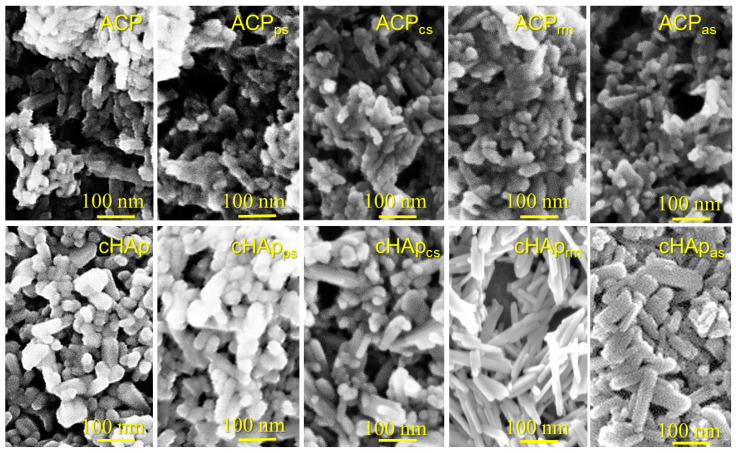
SEM micrographs of unloaded and STR-loaded ACP and cHAp nanoparticles.

Figure 2 shows representative micrographs for the different prepared hydroxyapatite nanoparticles. All the particles were prepared from a first-step precipitation reaction based on the addition of a calcium solution into a phosphate solution (see Section 3.2, Section 3.3 and Section 3.4). In addition, the amorphous (ACP) and crystalline (cHAp) samples were subsequently obtained by aging and thermal treatments, while different STR loading processes were considered. Figure 2 shows representative micrographs for the different prepared hydroxyapatite nanoparticles. All the particles were obtained from a first precipitation step based on the addition of a calcium solution into a phosphate solution (see Section 3.2, Section 3.3 and Section 3.4). In addition, amorphous (ACP) and crystalline (cHAp) samples were subsequently obtained by aging and thermal treatments, respectively, while different STR loading processes were considered. Note that ACP and cHAp correspond to different phases of calcium phosphate, as mentioned in the Introduction. On the other hand, STR was used as a template for particle nucleation due to the positive charge of the amino group (e.g., streptidine domain, Figure 1); consequently different interactions with phosphate and calcium ions were expected. Therefore, STR was loaded together with the calcium solution (cs) or the phosphate solution (ps). Both options represent the incorporation of STR into the mineral structure. The structural incorporation of STR in the early stage of precipitation (rm) is also possible and means the incorporation of STR into crystallites. These structural loading pathways for STR promote binding between the drug and the mineral to prevent drug leakage. By contrast, when the drug is adsorbed (as) onto the mineral, drug leakage can frequently occur.

Morphological differences were relatively scarce but can be summarized as follows: (a) The ACP nanoparticles featured smaller dimensions than the cHAp and showed higher agglomeration and more poorly defined morphologies. Thus, rounded particles with diameters lower than 20 nm were predominant in the ACP preparations, although some cylindrical particles with shorter than 70 nm could also be observed. By contrast, the cHAp particles mostly presented a nanorod morphology with diameters and lengths around 20–50 nm and 50–150 nm, respectively. Consequently, the applied hydrothermal process led to repercussions for the final morphology, as demonstrated by the crystalline structure. (b) The incorporation of STR did not exert a significant influence on the morphology of the ACP nanoparticles, while in general, the size of the loaded cHAp nanoparticles was increased. (c) The size of the STR-loaded cHAp nanoparticles was slightly affected by the way the drug was incorporated. Thus, bigger rods were attained when the drug was added into the reaction medium (i.e., cHAp_rm_ samples characterized by the incorporation of STR in the outer shell of the formed nanoparticles). STR appeared to favor the longitudinal growth of nanoparticles during the hydrothermal treatment, but also appeared to affect the morphology after a simple adsorption process on the crystalline surfaces (i.e., note the differences between the morphologies of the cHAp_as_ and the cHAp nanoparticles). Although the adsorption was an unspecific binding controlled by weak forces, such as electrostatic and hydrophobic interactions, the structural order of the substrate may have driven the adsorption process of the STR.

Table 1 reveals that the STR loading percentage (DL) was always higher for the cHAp than for the ACP nanoparticles independently of the incorporation method. This feature suggests that better interaction could be established using the molecularly ordered substrate, a feature that is in agreement with the morphological observations. Obviously, the incorporation of STR was the highest for the adsorption process (i.e., 3.2 and 3.6 *w*/*w*-% for ACP and cHAp, respectively). By contrast, the lowest amount of STR incorporated in the particles occurred when it was incorporated in the final phase of the synthesis, when the calcium had reacted with the phosphate. Finally, it was also notable that a significant amount of drug was incorporated when it was added from the calcium and phosphate solutions, but the values were higher for the second case (e.g., 2.2% with respect to 1.9% and 1.8% with respect to 1.4% for the cHAp and ACP nanoparticles, respectively). These results concerning the STR load are well explained considering the clearly higher encapsulation efficiency (EE) determined for the cHAp nanoparticles (Table 1). Furthermore, the incorporation of STR in the phosphate solution was more effective (i.e., 25.9% and 21.5%) than the loading from the calcium solution (i.e., 21.8% and 16.9%) or from the precipitation reaction medium (i.e., 7.7 % and 5.4%). The adsorptions onto the formed nanoparticles were only 13.2% and 11.7% for cHAp and ACP nanoparticles, respectively. Finally, it is interesting to note that the hydrothermal treatment of cHAp nanoparticles does not affect the chemical structure of STR, probably as consequence of the specific interactions with calcium and phosphate ions that provide thermal protection for STR during treatment. In fact, pure STR features a melting point of approximately 230 °C and its decomposition to form nitrogen oxides occurs at around 200 °C. The most labile is the sulfate STR form that corresponds to the unloaded STR in the nanoparticles; its melting point is 168 °C. This physicochemical characteristic of STR supposes in itself the resistance of the drug to hydrothermal treatment (e.g., 150 °C for 24 h at 200 atm) to produce cHAp.

**Figure 3:**
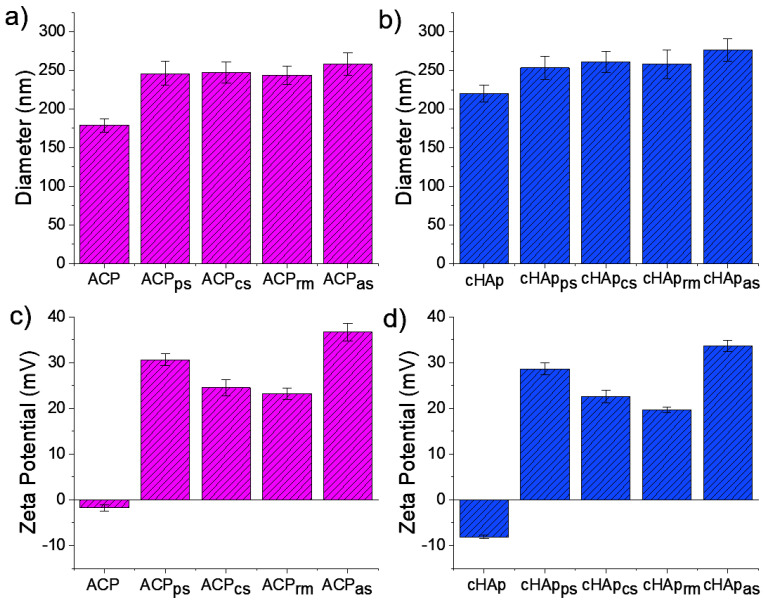
Hydrodynamic size (**a**,**b**) measured by dynamic light scattering (DLS) and Zeta-potential (**c**,**d**) for unloaded and STR-loaded ACP (**a**,**c**; red bars) and cHAp (**b**,**d**; blue bars) nanoparticles.

The hydrodynamic sizes of the ACP and cHAp nanoparticles unloaded and loaded with STR are summarized in Figure 3. In general, the DLS measurements indicate that all the studied particles were on the nanometric scale. The unloaded ACP nanoparticles featured the smallest sizes, with an effective diameter of 179 ± 30 nm, while the cHAp nanoparticles featured a greater effective diameter (i.e., 220 ± 38 nm). When the nanoparticles were loaded during synthesis, the effective diameter was increased and values close to 250 nm were determined independently of the type of particle and loading method. A significant increase was detected after adsorption, however; this increase was significantly higher for the cHAp_as_ particles (i.e., 258 ± 47 nm and 276 ± 51 nm for ACP_as_ and cHAp_as_, respectively) (Figure 3a,b).

The electrophoretic charge of the nanoparticles was determined according to the Zeta-potential method (Figure 3c,d). A negative charge of −8.1 ± 1.9 mV and −1.8 ± 3.9 mV were found for the cHAp and ACP nanoparticles, respectively. The great observed difference can explain the clear differences between the aggregations of the ACP and cHAp particles. The incorporation of STR always led to a net positive charge due to its intrinsic positive charge. The highest positive value was achieved by adsorption (i.e., 33.7 ± 6.1 mV and 36.7 ± 9.7 mV for ACP_as_ and cHAp_as_, respectively), followed by samples resulting from the addition of STR into the phosphate and calcium solutions. Finally, the incorporation of STR in the reaction medium was the incorporation that introduced the least positive charge (i.e., 19.7 ± 3.6 mV and 23.2 ± 6.1 mV for ACP_rm_ and cHAp_rm_, respectively). These changes in the Z-potential (i.e., the positive charge achieved after STR loading) appear to be in full agreement with the experimental STR-loaded doses.

**Figure 4:**
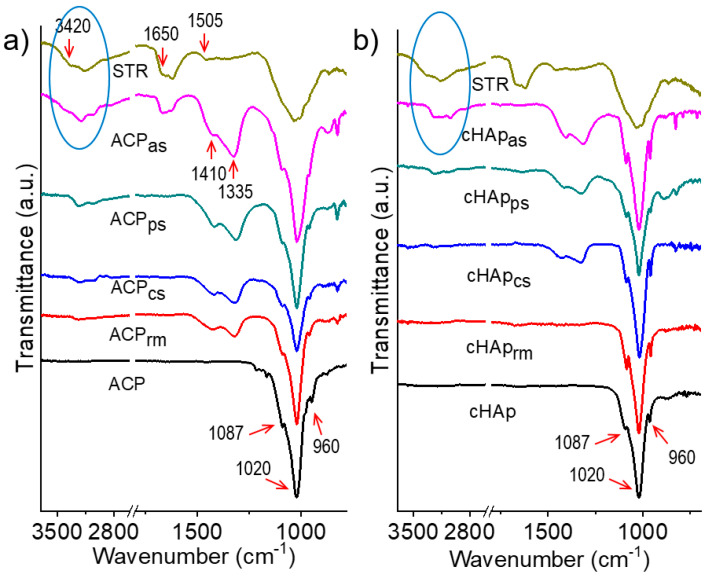
FTIR spectra of unloaded and STR-loaded ACP (**a**) and cHAp (**b**) nanoparticles.

FTIR spectra were recorded to qualitatively verify the incorporation of STR into the nanoparticles (Figure 4) without performing an extraction and release experiment. Thus, the characteristic bands of the (PO_4_)^3−^ groups at ca. 960, 1020 and 1087 cm^−1^ were clearly detected in the spectra of the nanoparticles. The profiles corresponding to the cHAp and ACP samples could be differentiated since the former presented more defined peaks (e.g., 960 and 1087 cm^−1^ peaks could be well distinguished in the profiles of cHAp, whereas they appeared as broad shoulders in the ACP spectra). In addition, (CO_3_)^2−^ absorption bands were detected in the spectra of the nanoparticles loaded with STR (see the broad doublet at 1335 and 1410 cm^−1^). The relative intensity of these signals was dependent on the incorporation method and the type of nanoparticle. In general, the relative intensity of the (CO_3_)^2−^ absorption bands was higher for the ACP-derived samples and specifically when STR was incorporated by adsorption. The FTIR spectra of STR, used as a control, showed vibrational bands at 3420 cm^−1^, 1650 cm^−1^ and 1505 cm^−1^, which could be assigned to the phenolic O-H group, the aromatic C=C double bond group and the amino (NH_3_^+^) group (see Figure 1). Essentially, phenolic and C=C bands could be observed in the nanoparticles with the highest loads of STR, namely those prepared by adsorption. Note that the intensity profiles of these bands were slightly different for pure STR and loaded samples (see ellipsoids in Figure 4), suggesting the ability to establish effective interactions between the drug and the inorganic compound through the OH groups of STR.

**Figure 5:**
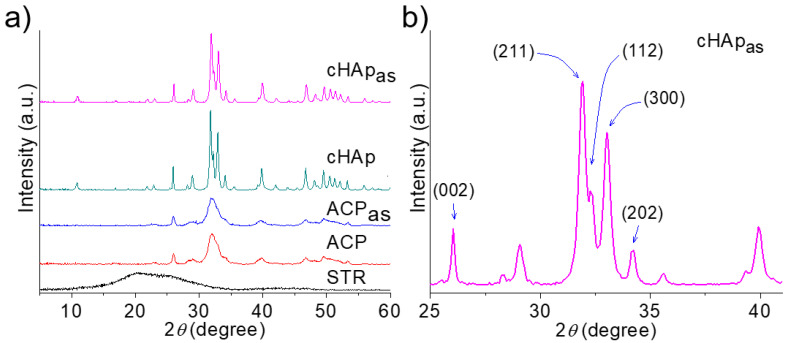
X-ray diffractograms of unloaded and STR-loaded nanoparticles and STR as a control (**a**). Indexed Bragg peaks for the cHAp_as_ sample (**b**).

The X-ray powder diffraction (XRD) patterns of the different nanoparticles are shown in Figure 5. The diffraction profiles were identical when the unloaded and STR-loaded samples were compared, even at the higher amounts of incorporated STR (i.e., the samples obtained by adsorption). In fact, the STR profiles where characterized by the presence of two amorphous halos at 0.434 and 0.346 nm, which were not relevant in the profiles of the nanoparticles due to the low incorporated STR amount. The cHAp profiles showed well-defined crystalline reflections, which could be univocally assigned to the inorganic nanoparticles at 25.9° (002), 31.8° (211), 32.2° (122), 32.9° (300) and 34.1° (202) (Figure 5b) (ICDD number 9-432). Namely, no additional peaks indicative of potential impurities could be detected. The ACP X-ray diffraction pattern showed very broad signals at similar spacings that reflected the lower crystal size attained after the simple aging process. The degree of crystallinity and crystalline size (determined according to Equations (1) and (2), respectively) decreased from 78.4% to 24.0% and from 28.3 nm to 11.8 nm, respectively. Table 2 also summarizes the crystallinities and crystal sizes of the samples after the incorporation of STR. Although the shape of the respective X-ray diffraction profiles remained practically unchanged, minor changes were detected in both the degree of crystallinity and the crystallite size after the STR incorporation.

These parameters were lower for the STR-loaded nanoparticles, suggesting some kind of interaction, as also suggested by the FTIR spectra (Figure 4), which could affect both the nucleation and the crystal growth rate. Note that STR features -OH and –NH groups in its streptodine (inositol with guanido groups) and streptoscamine (N-methyl-L-glucosamine) rings (Figure 1) that can form new hydrogen bonds and affect the structure and stability of both ACP and cHAp nanoparticles. This structural alteration was significantly higher when STR was added to the outer part of the nanoparticles before performing the aging and hydrothermal treatments. The adsorption process also influenced the final crystallinity and morphology, as indicated above.

**Figure 6:**
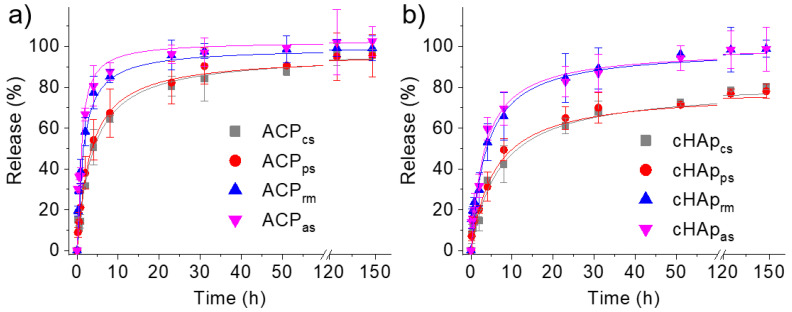
STR release profiles in PBS for ACP (**a**) and cHAp (**b**) nanoparticles loaded with STR according to the different studied methods.

The functional characterization of the synthesized nanoparticles was carried out by studying the release of the STR antibiotic (Figure 6). The results indicate that STR could be effectively released from both ACP and cHAp nanoparticles, although in all cases, the release rate was slower for the cHAp than for the ACP. On the other hand, the release rate was significantly slower for the nanoparticles when STR was loaded in the calcium and phosphate solutions. In these cases, STR could be incorporated in the inner parts of the nanoparticles and, therefore, the release was delayed and a regulating effect was achieved. The faster STR release for the loaded nanoparticles at the end of synthesis (ACP_rm_ and cHAp_rm_) and by adsorption (ACP_as_ and cHAp_as_) was attributed to the diffusion of the STR superficially encapsulated/adsorbed in the nanoparticles.

The release kinetics can be calculated from the experimental results by several theoretical models, typically first-order [32,33] and Higuchi [34,35] models. Drug release generally occurs in two steps: a rapid release of molecules, which should be mainly deposited on the high surface area of the nanoparticles; and a slow release, which should involve the diffusion of molecules through the nanoparticle bulk towards the outer medium. In this way, a combined model based on the two above models is usually used to describe the first (0–60%) and second (40–100%) parts of the release [36,37], as stated in Equation (1) (Higuchi model) and Equation (2) (first-order models):

(1) $$\begin{array}{cc} \frac{M_{t}}{M_{0}}=k_{H}t^{1/2} & \left(0\right.\leq \frac{M_{t}}{M_{0}}\leq \left.0.6\right) \end{array}$$

(2) $$\begin{array}{cc} \ln(1-\frac{M_{t}}{M_{0}})=a-k_{1}t & \left(0.4\right.\leq \frac{M_{t}}{M_{0}}\leq \left.1\right) \end{array}$$

where *k_H_* and *k*_1_ are the Higuchi and first-order release constants, respectively, *a* takes into account the release in the first step, *M_t_* is the percentage of the drug released at time *t* and *M*_0_ is the drug equilibrium percentage (considered as the maximum drug percentage).

The kinetic data are summarized Table 3. They make it possible to verify that ACP- and cHAp-loaded nanoparticles feature different release profiles in the PBS physiological medium. The fact that STR molecules feature several groups able to interact with calcium and phosphate and the slower diffusion rate through the matrix caused by the high molecular size are both fundamental. 

In the PBS medium (hydrophilic medium), the first part of the release was clearly slower for the cHAp nanoparticles. Smaller values were determined for the Higuchi constant, suggesting that a greater ratio of STR molecules was incorporated into the inner part of the nanoparticles. The release was delayed and a regulating effect was achieved. The faster STR release for the loaded nanoparticles at the end of synthesis (ACP_rm_ and cHAp_rm_) and by adsorption (ACP_as_ and cHAp_as_) was attributed to the easy diffusion of the STR superficially encapsulated/adsorbed in the nanoparticles. Values of *k_1_* indicate a very slow release at the end of the process. As expected, this release was again slower for the crystalline samples.

**Figure 7:**
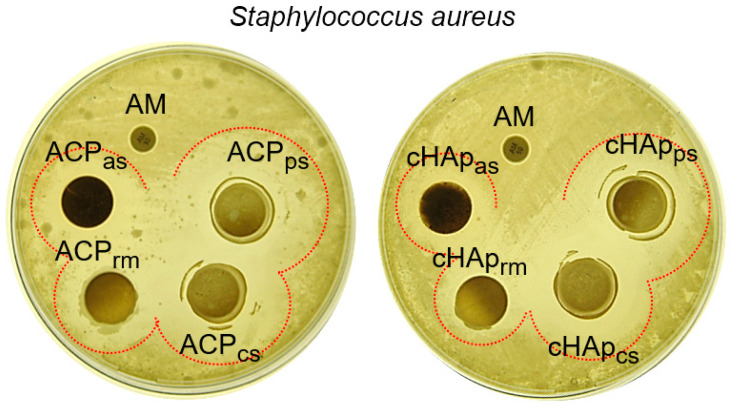
Inhibition of *Staphylococcus aureus* growth by STR. Antibacterial activity is shown by the formation of the inhibition halos; the bacterial strain is very sensible to STR. AM, commercial ampicillin disk. The dotted lines show the inhibition zones.

The antibacterial activity of the loaded STR was tested against *Staphylococcus aureus* as a representative Gram-positive bacteria. Figure 7 shows the inhibition halos achieved with the differently loaded ACP and cHAp nanoparticles. It should be noted that the antibiotic ampicillin (an β-lactam antibiotic that was selected as a “negative control”) rendered a practically negligible halo due to the resistance of the *S. aureus* strain used in the assay. By contrast, this strain was highly susceptible to STR. However, both kinds of nanoparticles showed a higher inhibition, especially when STR was loaded from the phosphate (ACP_ps_ and cHAp_ps_) and calcium (ACP_cs_ and cHAp_cs_) solutions. The lowest inhibition occurred when STR was loaded once a former mineral nucleus was obtained (e.g., when the STR was loaded immediately after the precipitation reaction). These results fit well with the lower STR load in this sample (Table 1). The relatively low effect observed with the STR-adsorbed samples seems contradictory considering both their higher pay load and their fast release. STR is an aminoglycoside antibiotic that contains cyclitol moieties, to which amino sugars are covalently linked through glycosidic bonds (Figure 1). These compounds are sensible to several biochemical reactions, including oxidation, reduction, hydrolysis and hydration [38]. Therefore, the STR adsorbed in the nanoparticles (ACP_as_ and cHAp_as_) can easily be biochemically altered (e.g., note the black color of the disk in the plate) leading to reduced antibacterial activity.

**Figure 8:**
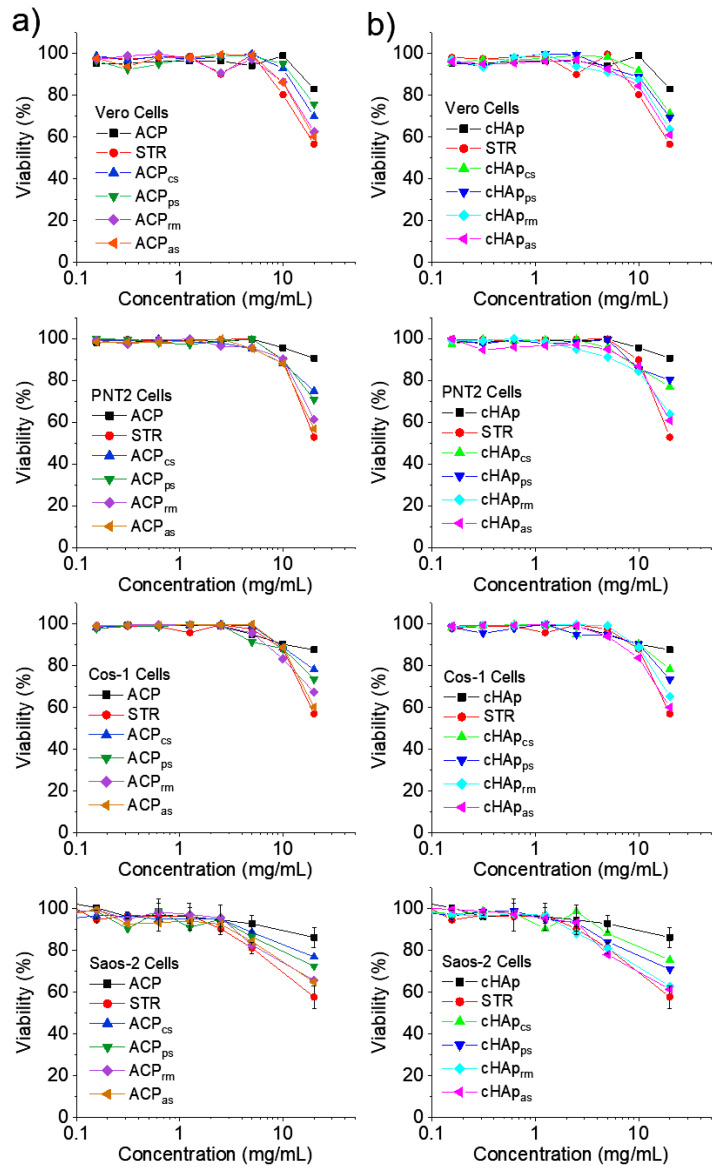
Cytotoxicity curves for ACP (**a**) and cHAp (**b**) nanoparticles.

The cytotoxic effect was assessed by means of examining the activity of the extracts prepared from both ACP and cHAp nanoparticles. The cytotoxic activity was examined via MTT assay against selected cell lines, namely Vero, PNT2, Cos-1 and Saos-2 cells (Figure 8). The results revealed that nanoparticle extracts at high concentration (e.g., 20 mg/mL) exert significant cytotoxic effects against the tested cell lines. The results clearly indicated that epithelial (Vero, PNT2, Saos-2) and fibroblast (Cos-1) cells were susceptible to highly concentrated nanoparticle extracts. In addition, no selectivity of the extract was observed against normal cell lines (Vero) and both cancer cells (Saos-2) and immortalized cells with SV40 (PNT2, Cos-1). The presence of STR in the extracts (as revealed by drug release and antibacterial experiments) may account for the observed cytotoxic properties. In this way, STR was used as a positive control in the experiments. Note that the historical development of the prophylactic use of STR as an antibiotic in cell culture as well as its effects on cells, has been reported [39]. The influence of STR on cell morphology, cellular degeneration, cellular function and cell death occurs because the protein synthesis is impaired. This feature can cause interference with, or even changes in, metabolic processes. Although STR and penicillin are widely used antibiotics under standardized conditions in cell cultures due to their low cytotoxicity, it should be considered that the cytotoxic behavior depends on the type of cell. In this way, low cytotoxicity is observed for leukocytes and macrophages, but a rather high cytotoxicity can be found in the case of fibroblasts [40].

FTIR microspectroscopy is a physicochemical analytical technique for identifying macromolecular changes that involves taking into account characteristic absorbance bands. In order to identify the band components, the second derivative of spectral peaks is analyzed. Specifically, three regions of the FTIR spectra of cells are considered: 3000–2800 cm^−1^ for lipids, 1700–1500 cm^−1^ for proteins and 300–900 cm^−1^ for carbohydrate and nucleic acid components. FTIR microspectroscopy is widely employed for the identification of bacteria on a general level [41], to discriminate cancer cells [42], to differentiate cell death by necrosis or apoptosis [43,44] and to point out the different metabolic states of cells [45,46].

Principal component analysis (PCA) provides a summary of the main information contained in tabulated multidimensional data, in which the FTIR spectra of Saos-2 cells (control and STR treated cells) are presented in rows, while variables (the wavenumbers within selected intervals) are presented in columns. When these are projected into a virtual bi-dimensional space, a smaller number of latent variables, called principal components (PC), can summarize most of the relevant information carried by the original variables. 

The first PC contains the greatest source of information in the data set and each subsequent PC contains less information than the previous one. By maximizing the dispersion of data points (each one corresponding to a given spectrum) in a multi-dimension virtual space, the PCA can be applied to (a) perform a multivariate explorative analysis allowing the identification of the most relevant variables by the unsupervised method and (b) separate sample groups (the spectra of control and STR treated cells).

**Figure 9:**
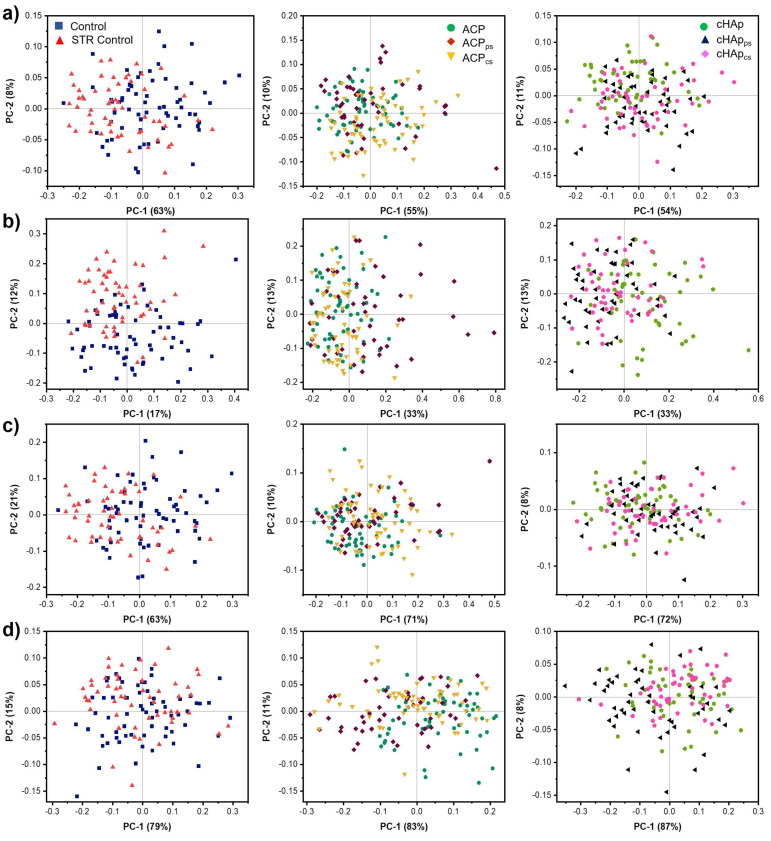

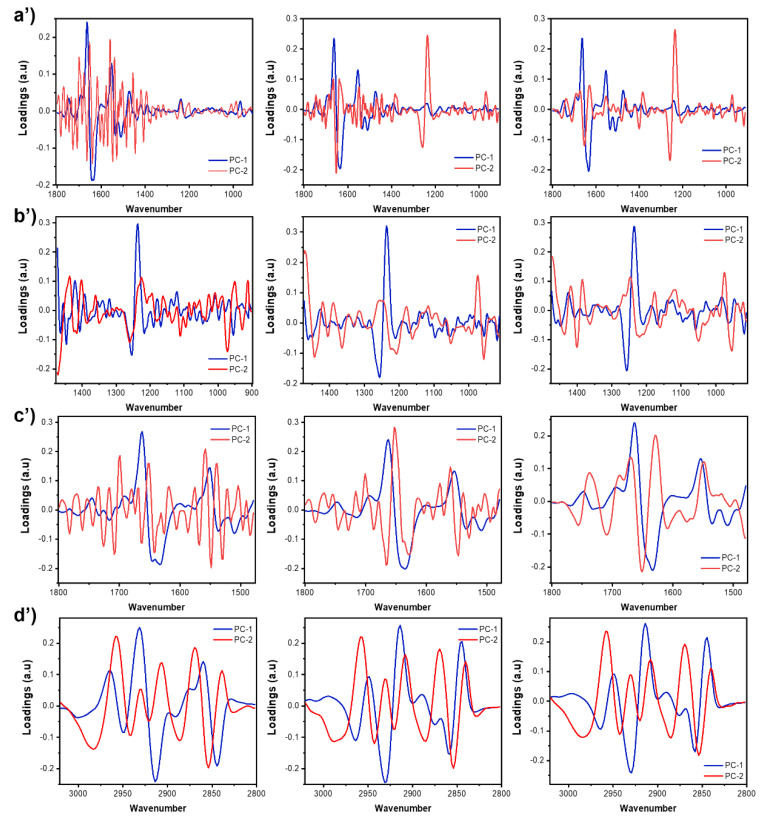
Principal component analysis (PCA) score plots (**a**–**d**) and loading plots (**a’**–**d’**) from 2nd derivative spectra of groups of treated Saos-2 cells. Biochemical changes from each biochemical group were discriminated considering the PC1 versus PC2 score plot: (**a**,**a’**) fingerprint, (**b**,**b’**) DNA and glucids, (**c**,**c’**) proteins and (**d**,**d’**) lipids.

Figure 9 illustrates the classifications of each data set analyzed by the PCA performed from the second derivative spectra of the cell samples. Two clusters of spectra from the control Saos-2 cells and STR-treated Saos-2 cells were clearly visualized in the two-dimensional score plots (PC1 vs. PC2 score plots) from the PCA modeling. The cluster of the STR-treated Saos-2 cells was separated from the cluster of control Saos-2 cells using PC1 (62%) and PC2 (8%) (Figure 9a). These results suggest that the Saos-2 cells with the STR treatment showed changes in their biomolecular components in comparison with the non-treated Saos-2 cells (control).

PCA is a conversion of FTIR peaks (the old coordinate system) into PC (the new coordinate system). Essentially, PCA indicates how much FTIR peaks (i.e., the loading values) contribute to each new PC. Regarding the second derivative in the region of carbohydrates and nucleic acids (Figure 9b), it was observed that the treated STR cells demonstrated a significant increase in the intensity of the bands associated with the phosphate and glycogen groups (i.e., the region between 1080 and 1030 cm^−1^) and a significant decrease in the bands corresponding to the vibration of the ribose phosphate backbone (992 cm^−1^) and the ribose ring (914 cm^−1^). Similarly, the STR-treated Saos-2 cells also showed a displacement and increase in intensity for the bands corresponding to the extension vibrations of the DNA skeleton (986 cm^−1^), as well as the spectral band at 899 cm^−1^ associated with the DNA/RNA. The results for the protein amide I band are displayed in Figure 9c. The analysis made it possible to differentiate between the components of the secondary structure of the proteins, such as β-turns (1693 cm^−1^ and 1682 cm^−1^), α-helices (1650 cm^−1^) and β-sheets (1634 cm^−1^). Compared with the control, the STR-treated Saos-2 cells showed an increase in the intensity of β-turn bands and a decrease in the intensity of bands corresponding to α-helices and β-sheets. Figure 9d presents the clustering of negative loading plots for the acyl lipid chain bands at 2958 cm^–1^ (ν_as_ CH_3_^−^), 2921 cm^–1^ (ν_as_ CH_2_^−^) and 2852 cm^–1^ (ν_s_ CH_2_^−^). The respective lipid content in the control Saos-2 cells group was, therefore, higher than in the STR-treated Saos-2 cells.

However, the cluster separation of the cells treated with hydroxyapatite and STR-loaded hydroxyapatite nanoparticles was more complex, particularly for cHAp nanoparticles. By contrast, the ACP nanoparticles presented clear evidence of the separation of some clusters when STR was loaded in the Ca^2+^ and in the (PO_4_)^3−^ components of the nanoparticle.

### 2.2. Preparation of Electrospun PLA Matrices Loaded with Nanoparticles Incorporating STR

Electrospun PLA fiber matrices have been prepared and applied as temporary scaffolds for tissue engineering and repair. The incorporation of biomaterials such as ACP and cHAp nanoparticles increases their biodegradation due to the mineralization of the fibers. Furthermore, the great hydrophilicity of these nanoparticles allows the incorporation of hydrophilic drugs in the bulk of the fibers. In this study, ACP_ps_ and cHAp_ps_ nanoparticles were incorporated into PLA fibers during their electrospinning from CHCl_3_:acetone (2:1 *v/v*) suspensions with 2.5 %-*w*/*v* of the corresponding nanoparticles and 10%-*w*/*v* of dissolved PLA. The phosphate-loaded nanoparticles were selected considering the amount of loaded STR, the relatively fast release of STR and the greater antibacterial effect. The ACP_as_ and cHAp_as_ nanoparticles were not considered for electrospinning due to the fact that the binding of STR to the nanoparticle matrix is non-specific. Furthermore, there was the possibility that the STR would be damaged due to its reduced antimicrobial activity, as previously demonstrated.

**Figure 10:**
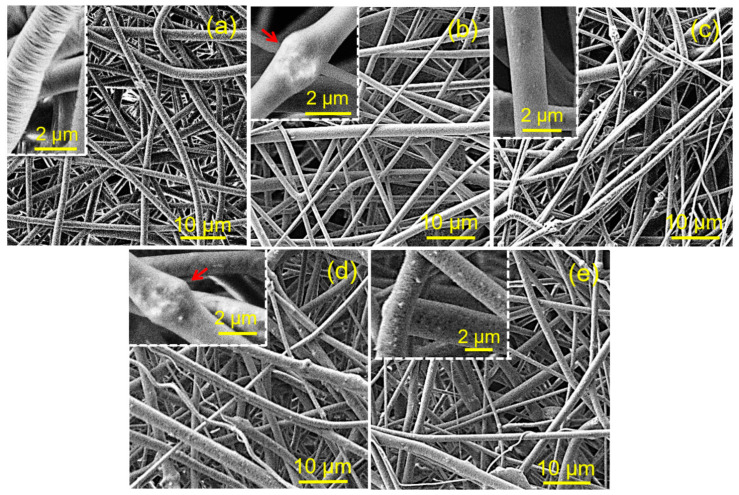
SEM micrographs of electrospun PLA fibers (**a**), PLA/ACP (**b**), PLA/ACP_ps_ (**c**), PLA/cHAp (**d**), PLA/cHAp_ps_ (**e**). Nanoparticles aggregates are pointed out with yellow arrows. Insets show magnified images. Arrows show nanoparticles aggregates.

Figure 10 shows the morphology of the PLA fibers containing STR-loaded nanoparticles. In general, at low magnification, the fibers were uniform and featured an homogeneous diameter distribution. However, some morphological peculiarities can be highlighted when observations are carried out at higher magnification. The PLA fibers showed porous surfaces with transversal striations (inset of Figure 10a). Nodules of nanoparticle aggregates were observed in the PLA fibers loaded with ACP and cHAp (Figure 10b,d). By contrast, these nodules were absent when the ACP_ps_ and cHAp_ps_ nanoparticles were incorporated. In fact, in these cases, the surfaces of the fibers showed fine porosity and a regular distribution of nanoparticles. Agglomeration seems to have been avoided, probably as a consequence of the increase in the Zeta potential after the STR load.

**Figure 11:**
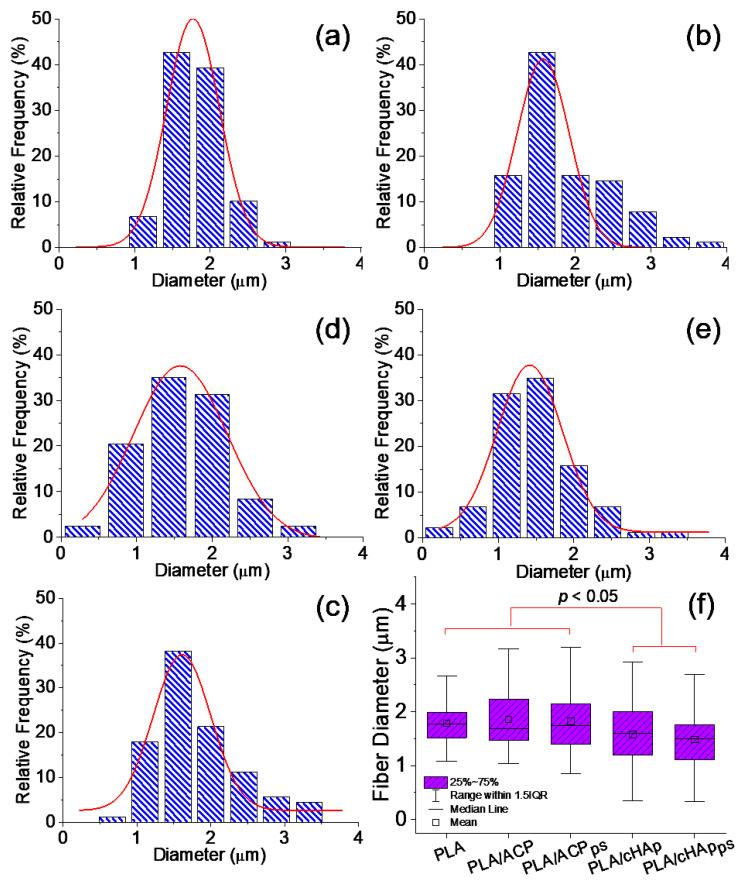
Distribution of fiber diameters for electrospun PLA fibers (**a**), PLA/ACP (**b**), PLA/ACP_ps_ (**c**), PLA/cHAp (**d**), PLA/cHAp_ps_ (**e**). Box plot for fiber diameter measurements (**f**); *p* < 0.05 by ANOVA-Tukey test.

The diameters of the electrospun fibers consistently showed a Gaussian distribution (Figure 11a–e), with values in the micrometric range and scarce differences. However, Figure 11f reveals that the average fiber diameter was significantly smaller in the PLA/cHAp and PLA/cHAp_ps_ matrices. Thus, values of 1.58 ± 0.57 µm (PLA/cHAp) and 1.49 ± 0.52 µm (PLA/cHAp_ps_) were obtained, in contrast with the values of 1.86 ± 0.58 µm and 1.82 ± 0.59 µm that were measured for the PLA/ACP and PLA/ACP_sp_ matrices, respectively. These values were practically identical to that determined for the neat to PLA matrix (1.79 ± 0.34 µm). Probably, the nanorod morphology of the crystalline nanoparticles (cHAp and cHAp_ps_) contributed to the longitudinal orientation in the PLA matrix and, consequently, the fibers showed a reduced diameter compared to the fibers containing the amorphous nanoparticles (ACP and ACP_ps_).

**Figure 12:**
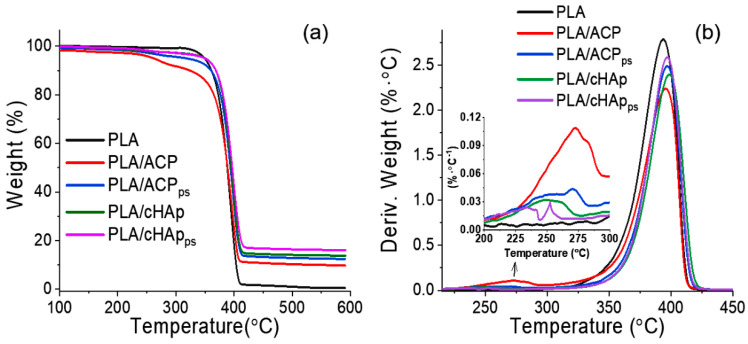
TGA (**a**) and DTGA (**b**) curves for PLA scaffolds incorporating unloaded and STR-loaded nanoparticles of amorphous ACP and crystalline cHAp hydroxyapatite.

The effective incorporation of inorganic nanoparticles was also determined through thermal degradation analysis, taking into account the remaining weight after heating the sample at temperatures higher than 500 °C. This residual weight mainly corresponds to the presence of the mineral incorporated in the fibers (Figure 12a), the main values being around 18%, as expected from the theoretical load (i.e., 20%). The degradation curves of samples incorporating hydroxyapatite showed a minor first decomposition step around 300–320 °C, which was higher for the ACP-loaded samples and could be associated with impurities (e.g., carbonates). These carbonates were probably mostly eliminated after the hydrothermal treatment applied to obtain cHAp and in fact were not present in the neat PLA matrices. The DTGA curves (Figure 12b) clearly evidence a shift towards higher temperatures and close to 10 °C for the main degradation peak (e.g., from 380 °C to 390 °C) when ACP or cHAp particles were incorporated, suggesting a protective effect on the degradation of PLA. Minor differences were detected for the samples with nanoparticles with encapsulated STR, although a slight increase in the residual weight was observed for both matrices incorporating ACP or cHAp. 

**Figure 13:**
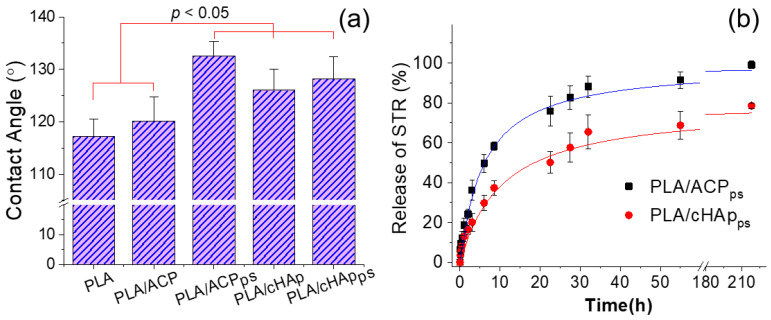
Contact angles (**a**) and STR release curves in a PBS medium (**b**) determined for different PLA electrospun matrices.

The surface properties of the different PLA scaffolds were evaluated by measuring the contact angles of the water drops (Figure 13a). The results demonstrate that the contact angle of the fibers containing the nanoparticles loaded with the hydrophobic STR (i.e., PLA/ACP_ps_ and PLA/cHAp_ps_) significantly increased (i.e., from 120–126° to 128–132°). Furthermore, the incorporation of nanoparticles slightly increased the contact angle of the neat PLA matrix (i.e., from 117° to 120–126°). This change may have been a consequence of the protuberances caused by the agglomeration of the nanoparticles, which were more abundant in the ACP-derived matrices (i.e., contact angle of 126°).

The hydrophobicity of the fibers can explain the slow and prolonged release of STR from the PLA fiber matrices containing loaded ACP and cHAp nanoparticles (Figure 13b). Note that a significant release (70–90%) was achieved in all cases despite the difficult process that implies the diffusion of the drug outside the encapsulating agent and the PLA microfiber. The release was faster when the drug was encapsulated in the more amorphous ACP nanoparticles, a feature that is in agreement with the more efficient entrapment of STR inside the crystalline cHAp domains. Note also that differences in porosity may exert a significant influence on the release [47]. The release was well simulated with the combined model (i.e., Higuchi model for 0–60% of release and first-order model for 40–100% of release). Table 4 summarizes the corresponding release constants. Note that the release of fibers loaded with the amorphous ACP nanoparticles was the fastest, as deduced from the values of the kinetic constants.

## 3. Materials and Methods

### 3.1. Materials

Streptomycin sulfate salt powder (C_21_H_39_N_7_O_12_ 1.5H_2_SO_4_), calcium nitrate tetrahydrate (Ca(NO_3_)_2_·4H_2_O), diammonium hydrogen phosphate ((NH_4_)_2_HPO_4_), and ammonium hydroxide solution 30% (NH_4_OH) were purchased from Sigma-Aldrich (Saint Louis, MO, USA). Chloroform stabilized with amylene (50 ppm), ethanol and acetone were provided by Fisher Scientific S.L. (Madrid, Spain). All chemicals were of analytical grade and were used without further modification and purification.

Polylactide (PLA) was obtained from Natureworks (Minnetonka, MN, USA) with 95.8 *wt*-% of L-lactic isomer (PLA 2002D) in a transparent pellet with a density of 1.24 g/cc. Its calorimetric and mechanical properties are defined by a glass transition temperature (*T_g_*) of 58 °C, a melting point (*T_m_*) of 153 °C, a tensile modulus (*E*) of 3500 MPa, a tensile strength (*σ*) of 53–60 MPa and a tensile elongation (*γ*) of 6%. Molecular weights determined by GPC were 98,100 g/mol, 181,000 g/mol for *M_n_*, *M_w_*, respectively and the polydispersity index was 1.85. 

A bacterial strain of *Staphylococcus aureus* was obtained from the Spanish collection of type culture (CECT, Valencia, Spain) and used in this study. This bacteria is a human pathogen of clinical significance, since it is responsible for a range wide of minor-to-life-threatening infections.

Vero (epithelial cell derived from normal kidney of *Cercopithecus aethiops*, “African green monkey”), Cos-1 (fibroblast-like cell immortalized with SV40, derived from normal kidney of *Cercopithecus aethiops*, “African green monkey”), Saos-2 (epithelial-like cell derived from human osteosarcoma) were obtained from American Type Culture Collection (ATCC, Manassas, VA, USA). PNT2 (cell line human, normal prostate epithelium immortalized with SV40) was obtained from European Collection of Authenticated Cell Culture (ECACC, Merck, UK).

### 3.2. Synthesis of Amorphous (ACP) and Crystalline (cHAp) Hydroxyapatite

A total of 5.91 gr of Ca(NO_3_)_2_ 4H_2_O was dissolved in 33.4 mL ethanol absolute and the pH was adjusted to 11 with NH_4_OH solution (30 *wt*-%). This solution was dropwise added to a second solution of 1.98 gr of (NH_4_)_2_HPO_4_ in 20 mL of ultrapure water (MilliQ, Millipore™, Darmstadt, Germany). The addition was performed under agitation (400 rpm) at a rate of 2 mL/min and at room temperature. Subsequently, the reaction mixture was stirred for an additional hour under the same conditions. Next, in the case of amorphous hydroxyapatite (ACP), the final suspension was aged at rest for 24 h at 37 °C. For crystalline hydroxyapatite (cHAp), the solution was submitted to a hydrothermal process using an autoclave that operated at a pressure of 200 atm and a temperature of 150 °C during 24 h. Both ACP and cHAp precipitates were separated by centrifugation and washed sequentially twice with ultrapure water and then with ethanol 96°. The product was frozen for about 2 h before freeze-drying for 2 days. Finally, a white powder was obtained in both cases.

### 3.3. Hydroxyapatite Nanoparticles with Encapsulated STR

Three different procedures were tested to encapsulate STR into ACP and cHAp samples: (i) addition of 1.5 g of STR into the (NH_4_)_2_HPO_4_ solution; (ii) addition of 1.5 g of STR into the Ca(NO_3_)_2_ solution; and (iii) addition of 1.5 g of STR into the mixture of (NH_4_)_2_HPO_4_ and Ca(NO_3_)_2_ (i.e., STR should be incorporated after the preliminary formation of the precipitate). The corresponding nanoparticles were named as ACP_cs_, ACP_ps_ and ACP_rm_ to indicate that STR was loaded in the amorphous phase (ACP) from the calcium solution (cs), the phosphate solution (ps) and the reaction mixture (rm), respectively. Crystalline nanoparticles were obtained from the same three procedures, but logically the above indicated final hydrothermal treatment was applied at the last step. In fact, pure STR features a melting point of approximately 230 °C and its decomposition to form nitrogen oxides occurs at around 200 °C. This physicochemical characteristic of STR supports its resistance during hydrothermal treatment. The different nanoparticles were named cHAp_cs_, cHAp_ps_ and cHAp_rm_ (c for crystalline particle), according to the previous abbreviations. 

STR concentration was determined by UV-Vis spectroscopy by reading at 280 nm and using a UV3600 Shimadzu model (Shimadzu Corp., Kyoto, Japan). Specifically, HAp particles were incubated in PBS-ethanol 70 %-*v/v* to remove the STR content in the shell or the surface layer. Next, the same particles were dissolved in 100 mM HCl and 50 mM NaCl solution to determine the STR loaded in the particle core or bulk. Quantification was performed using a standard curve at different STR concentrations (i.e., from 0.05 mM to 15 mM).

### 3.4. Adsorption of STR onto ACP and cHAp Nanoparticles 

A total of 250 mg of ACP or cHAp was introduced into 600 µL of a 154 mM STR solution. The corresponding suspension was maintained under agitation during 24 h at 25 °C. Sediments were resuspended in ultrapure water by centrifugation at 6500 rpm for 5 min at 4 °C and adducts were separated. After this process, which was repeated two times, the obtained pellets were frozen at −80 °C for 3h and, subsequently, the humidity was removed using a liophilizer. Finally, STR concentration was measured by UV-Vis spectroscopy, as described above. These particles were named ACP_as_ and cHAp_as_ (i.e., ACP or cHAp for amorphous or crystalline particles and the subscript *as* for the solution adsorption process).

### 3.5. Electrospun PLA Microfibers Loaded with HAp Nanoparticles

PLA microfibers were prepared by the electrospinning technique. Specifically, PLA (1 g) was dissolved in 10 mL of CHCl_3_:acetone (2:1 *v*/*v*) mixture (i.e., a polymer concentration of 10 *w*/*v*-%). The solution was kept under agitation (100 rpm) at 37 °C. Electrospinning was performed using a previously optimized setup [48] with a plane collector and a vertical disposition. After an optimization process where different experimental conditions were tested, 15 KV, 3 mL/h and 20 cm were selected for the electrostatic field, flow rate and tip-collector distance, respectively. An 18G needle with an internal diameter of 0.84 mm was used. The mats were accumulated on an aluminum foil sheet to facilitate their manipulation.

The different unloaded and STR-loaded nanoparticles (0.25 g) were added to the PLA solution and stirred for 1 h. The nanoparticle content in the electrospinning solution was 25 *wt*-%. It was fundamental to avoid sedimentation of the nanoparticles during electrospinning and, consequently, a peristaltic pump was employed while keeping the suspension under agitation. The mats of prepared microfibers were named indicating the matrix (PLA), the type of incorporated nanoparticle (ACP or cHAp) and the way the STR was incorporated (i.e., ps, cs, rm or as) as a subscript. For example, PLA/ACP_ps_ means a PLA scaffold loaded with amorphous nanoparticles in which STR was incorporated during synthesis with the phosphate solution. 

### 3.6. Characterization

#### 3.6.1. Scanning Electronic Microscopy (SEM)

SEM observations and chemical composition analyses were performed using high-resolution SEM equipment (Focus Ion Beam Zeiss Neon 40 Microscopy, Carl Zeiss, Germany) operating at an accelerating voltage of 5 kV. Samples were deposited on a silicon disc mounted with silver paint on pin stubs of aluminum and sputter-coated (Mitec K950 Sputter Coater) with a thin layer of carbon to prevent sample charging problems. An integrated energy-dispersive X-ray spectroscopy analyzer (EDX) was used to determine the atomic composition of the samples.

#### 3.6.2. UV-Vis Spectroscopy Analysis

A UV-Vis/NIR spectrophotometer (Shimadzu UV-3600 model, Tokyo, Japan) controlled with UVProbe 2.31 software was used to record the UV-Vis spectra at room-temperature. Scans were performed in the 200–400 nm range with a bandwidth of 0.2 nm and a scan speed of 600 nm/min. Samples were homogenized before recording the spectra.

#### 3.6.3. Fourier Transform Infrared (FTIR) Spectroscopy

The FTIR absorption spectra of the samples were recorded with a Fourier Transform FTIR 4100 Jasco spectrometer (Japan) in the 1800–700 cm^−1^ range. A Specac model MKII Golden Gate attenuated total reflection (ATR) machine with a heated Diamond ATR Top-Plate was used. 

#### 3.6.4. Dynamic Light Scattering (DLS) and Z-Potential

DLS and Z-potential were applied to measure the particle size and charges of hydroxyapatite nanoparticles. A NanoBrook Omni Zeta Potential Instruments from Brookhaven Instruments (Holtsville, NY, USA) was used. The Z-potential measured the charge of the interface between the solid surface and its liquid medium. Thus, 20 mg of hydroxyapatite nanoparticles was added to 20 mL of ultrapure water (MilliQ water, Millipore) and sonicated by 15 min at 45 % of power using an ultrasound (Sonopuls model, Bandelin, Berlin, Germany). DLS measurements consisted of three runs of 120 s duration that were subsequently averaged to give the effective diameter (Deff). Samples were analyzed at 25 °C, at a scattering angle of 173°. In order to determine the Z-potential, 0.4 mL of homogenized particles were resuspended in 3.6 mL of 1 M KCl solution and 30 consecutive measurements were performed for each sample.

#### 3.6.5. Wettability

Static contact angle measurements were recorded according to the sessile drop method and analyzed at room temperature. An OCA-15EC contact angle meter from DataPhysics Instruments GmbH (Fiderstadt, Germany) with SCA20 software (Version 4.3.12 build 1037) was employed. The sessile drop (MilliQ water) was gently placed on the surface of a matrix of electrospun PLA fibers using a micrometric syringe with a metallic needle (Hamilton 500 μL). The ellipse method was used to fit a mathematical function to the measured drop contour. For each PLA matrix, no less than 10 drops were examined.

#### 3.6.6. Thermal Degradation

Thermal stability of samples was studied at a heating rate of 20 °C/min with around 5 mg using a Q50 Thermogravimetric Analyzer of TA Instruments and under a flow of dry nitrogen. Test temperatures ranged from 50 °C to 600 °C.

#### 3.6.7. X-ray Diffraction (XRD)

Crystallinity was studied by wide angle X-ray diffraction (WAXD). Patterns were acquired using a Bruker D8 Advance model with Cu Kα radiation (λ = 0.1542 nm) and geometry of Bragg–Bretano, theta-2 theta. A one-dimensional Lynx Eye detector was employed. Samples were run at 40 kV and 40 mA, with a 2θ range of 10–60°, measurement steps of 0.02° and time/step of 2–8 s. Diffraction profiles were processed using PeakFit v4 software (Jandel Scientific Software) and the graphical representation was performed with OriginPro 2018 (64 bit) software (OriginLab Corporation, Northampton, MA, USA).

The crystallite size (L) in the direction perpendicular to representative (211) planes of cHAp was derived from the X-ray diffraction line broadening measurement using the Scherrer equation [49]:

(3) $$L=\frac{0.9\;\lambda }{\beta \;\mathrm{Cos}\;\theta }$$

where λ is the CuK_α_ wavelength, β is the full width at half maximum height of the (211) line, θ is the diffraction angle and 0.9 is a shape factor. 

The crystallinity (χ_c_) was obtained according to Equation (4) [50]:

(4) $$\chi_{c}=1-\frac{V_{112/300}}{I_{300}}$$

where I_300_ is the intensity of the (300) reflection and V_112/300_ is the intensity of the hollow between the (112) and (300) reflections, which disappears in non-crystalline samples.

### 3.7. Encapsulation or Adsorption Efficiency

Encapsulation or adsorption efficiency of ACP and cHAp samples were determined by spectrophotometry. STR-loaded nanoparticles (20 mg) were dissolved with 200 µL of 100 mM HCl and 50 mM NaCl mixture. Next, STR was extracted with 1 mL of phosphate buffered saline (PBS)-ethanol solution (PBS-EtOH, 70 %-*v*/*v*). Extraction medium was separated by centrifugation (Sigma 30K refrigerated centrifuge; Sigma-Aldrich Co, St Louis, MO, USA) at 10,000 rpm at 4 °C for 10 min. The amount of STR (experimental drug loading) was quantified by UV-Vis spectroscopy as indicated above and calculated as the difference between the total amount of drug used to prepare the loaded nanoparticles and that recovered by PBS-ethanol extraction.

Entrapment efficiency (EE) and drug loading (DL) were determined by Equations (5) and (6), respectively.

(5) $$\mathrm{EE}\left(\%\right)=\frac{\mathrm{Mass}\;\mathrm{of}\;\mathrm{drug}\;\mathrm{loaded}}{\mathrm{Mass}\;\mathrm{of}\;\mathrm{the}\;\mathrm{drug}\;\mathrm{in}\;\mathrm{the}\;\mathrm{synthesis}}\times 100$$

(6) $$\mathrm{DL}\;\left(\%\right)=\frac{\mathrm{Mass}\;\mathrm{of}\;\mathrm{drug}\;\mathrm{in}\;\mathrm{particles}}{\mathrm{Mass}\;\mathrm{of}\;\mathrm{particles}}\times 100$$

### 3.8. Release of Streptomycin from Loaded Nanoparticles

A total of 100 mg of each nanoparticle preparation was suspended into 50 mL of PBS at pH 7.4 as a physiological medium. Nanoparticles were confined in a dialysis bag to keep their mass constant during the release experiment. A quantity of the medium solution (1 mL) was removed for analysis at given time intervals and replaced with 3 mL of fresh release solution. The cumulative amount of streptomycin released into the solution was measured at different time intervals using a UV-visible spectrophotometer at 280 nm, as previously indicated.

### 3.9. Antibacterial Activity

The bacterial cultures were maintained in Luria-Bertani (LB) broth (Scharlau, Spain). Prior to incubation with the novel particles, the bacteria were cultured overnight in 10 mL of LB broth in a shaker at 37 °C and 120 rpm until the culture reached an OD_600_ = 1.0, which corresponds to 10^9^ colony-forming units per mL (CFU/mL). The overnight cultures were diluted to 10^8^ CFU/mL using sterile LB broth. The antimicrobial activity of the free STR (control) and loaded particles was evaluated against bacteria using the agar diffusion (disk-and-cup) method, as reported elsewhere [51]. Briefly, 20 mL of LB agar (pH 7.3 ± 0.2 at 65 °C) was poured onto disposable sterilized Petri dishes and allowed to solidify. The surface of the solidified agar plates was allowed to dry in an incubator prior to spreading the microorganisms onto their surface. Next, 100 µL of the microbial culture suspension in broth containing approximately 10^6^ CFU/mL, as measured spectrophotometrically, was streaked over the dried surface of the agar plate, spread uniformly using a sterile plastic rod and allowed to dry before applying the loaded samples. Nanoparticles (40 mg) were molded as disks (13 mm of diameter) using a press (5 Ton) and then were sterilized with UV light for 15 min. The STR dissolution (control) was loaded in a paper disk. The different loaded disks were applied carefully onto the surface of the seeded agar plates using sterile forceps. The zones of inhibition were evaluated after 24 h of incubation at 37 °C. 

### 3.10. Cytotoxicity Evaluation

The biocompatibility of the nanoparticle samples was evaluated in vitro by an extract cytotoxicity test according to ISO 10993-5, with modifications to prevent microbial contamination. The nanoparticles were extracted in cell culture medium and the extract was then evaluated in 96 well tissue culture plate. Cells were examined at 24 h of culture for signs of cytotoxicity. The nanoparticle extract was prepared according to the following procedure: nanoparticles (100 mg) were incubated in culture medium (5 mL) using sterile tubes of 15 mL for 24 h under culture conditions (37 °C and 5% CO_2_) and sterile conditions. After incubation, the tubes were centrifuged at 5000 rpm for 10 min. The culture media were recovered aseptically, filtered using a 0.22 µm syringe filter and finally stored at −20 °C until evaluation.

Vero, PNT2, Cos-1 and Saos-2 cells were cultured in Dulbecco’s modified Eagle’s medium (DMEM with 4500 mg/L of glucose, 110 mg/L of sodium pyruvate and 2 mM of L-glutamine) supplemented with 10 %-*v/v* fetal bovine serum (FBS), 100 U/mL penicillin, 100 µg/mL streptomycin and L-glutamine 2 mM at 37 °C in a humidified atmosphere of 5% CO_2_ and 95% air. Culture media were changed every two days. For sub-culture, cell monolayers were rinsed with PBS and detached by incubation with 0.25% trypsin/EDTA for 2–5 min at 37 °C. The incubation was stopped by resuspension in 5 mL of fresh medium and the cell concentration was determined by counting with a Neubauer camera and using 4% trypan blue as vital dye.

The 1 × 10^4^ cells were seeded in 96 well plates for 24 h to allow adhesion. Next, the culture media were removed by aspiration and replaced by serial dilutions of the nanoparticle extract prepared in fresh DMEM, as described above. These dilutions were in the range of 1/2 to 1/128 (*v/v*). The assay controls consisted of cells not exposed to the extracts (reference for 100% cell viability), extract of nanoparticles without the STR load and a positive control based on an STR solution (10 mg/mL). Cultures were maintained for 24 h and then the cell viability was determined by the MTT assay [52]. Culture media were aspirated and cells were washed twice with PBS. Subsequently, 100 µL of culture medium supplemented with MTT reagent at a final concentration of 1 mg/mL was added to each well. Plates were incubated for 3 h (during this time, viable cells converted the MTT reagent to insoluble formazan salts). The medium was then aspirated and 100 µL of DMSO was added to each well to dissolve the formazan salts and determine viability by measuring absorbance at 570 nm. The experiments were performed in triplicate and the values averaged.

### 3.11. FTIR Microspectroscopy

Saos-2 cells were directly exposed to the ACP and cHAp nanoparticles loaded with STR. Nanoparticles without the STR load were evaluated as controls in addition to the cells without any treatment. The cells were cultured in the presence of the nanoparticles at a concentration of 10 mg/mL for 24 h, according to the protocol previously described by Rivas et al. [53]. Preparation of cells for FTIR analysis was performed according to the procedure of Yousef et al. [54]. Cells were trypsinized and centrifuged at 540× *g* for 5 min, washed twice with 0.9 *w/v*-% NaCl, resuspended in 50 μL of NaCl solution, fixed with 2.5% formalin and finally washed with a NaCl physiological solution. The resuspended cells were dropped onto a CaF_2_ optical slide (13 mm diameter, 0.5 mm thickness) and then vacuum-dried for 30 min in a desiccator. The cells on the slide were gently rinsed for a few seconds with distilled water and then vacuum-dried. To completely remove the salt, this step was repeated. The washed and dried cell monolayer was stored in a desiccator until analysis. It should be noted that a quick rinse with distilled water was performed in order to remove the excess precipitated NaCl that was found all over the sample probing area. This salt content may have affected the variability of the non-homogeneous distribution of the cells. 

Cell fixation has been used in many studies to keep the structural biochemical constituents of the cells as close as possible to in vivo conditions. Formalin or any other organic compounds were, however, reported to feature strong absorption bands in the mid-infrared region causing interference to (or changes in) the infrared spectra of the cell sample [55]. Therefore, histological processing with 0.9 %-*w/v* NaCl is a standard technique used in FTIR studies since, it does not feature peaks in the range of the acquired IR spectra [56].

FTIR microspectroscopy was conducted to determine biomolecular changes in Saos-2 cancer cells. A Bruker Hyperion 2000 microscope (Bruker Optics Inc., Ettlingen, Germany) equipped with a nitrogen-cooled MCT (HgCdTe) detector (area 250 × 250 μm^2^) with a 36 × IR objective condenser, coupled to the Bruker Vertex 70 spectrometer, was used for IR data acquisition. IR signals were acquired from 70 to 100 cells within the 7 × 7 μm spot areas and mapped within the most homogeneous zones. The IR spectra were obtained from the transmission mode by collecting 64 scans at a resolution of 4 cm^−1^ over a measurement range of 3800–900 cm^−1^. This way, average spectra with an appropriate signal-to-noise ratio could be obtained. FTIR microspectroscopy was performed at the MIRAS beam line located at the ALBA Synchrotron Radiation (Cerdanyola del Vallès, Barcelona, Spain).

### 3.12. FTIR Microspectra Data Analysis

Principal component analysis (PCA) within spectral ranges of 1380–1000 cm^−1^ (phosphates), 1800–1477 cm^−1^ (amide I and II), 1760–1730 cm^−1^ (carbonyl group) and 3020–2800 cm^−1^ (lipids) was performed using Unscrambler 9.7 software (CAMO, Oslo, Norway). Data manipulations of FTIR spectra were adjusted by taking the vector-normalized second derivative (Savitzky–Golay algorithm, third polynomial) with 15 point smoothing, which allows the minimization of the effects of various baselines. This spectra normalization process accounts for differences in sample thickness and corrects scattering artifacts. Two PCs (PC1 and PC2) were considered for classification analysis. They were clearly separated for the different cell sample groups and were useful for interpretation. Data for each PC were plotted to visualize the clustering differences between cells [57].

### 3.13. Statistical Analysis

Values were averaged and graphically represented, together with their respective standard deviations. Statistical analysis was performed by one-way ANOVA test to compare the means of all groups. Next, Tukey test was applied to determine a statistically significant difference between two groups. The test confidence level was set at 95% (*p* < 0.05). For the graphical and statistical analysis, OriginPro 2018 (64 bit) software (OriginLab Corporation, MA, USA) was used in this study. 

## 4. Conclusions

STR-loaded ACP and cHAp nanoparticles can be successfully prepared in various stages of hydroxyapatite synthesis by using the precipitation method. The antibiotic was incorporated with a calcium component, with the phosphate group, during nucleation and adsorbed onto the nanoparticle formed. The best encapsulation efficiency was achieved when STR was added with the phosphate group component (22% and 26% for ACP and cHAp, respectively), followed by calcium (17% and 22% for ACP and cHAp, respectively). These efficiencies made it possible to obtain STR loads in the range of 1.4–2.2%. Smaller amounts of STR were encapsulated from the reaction mixture. The highest STR load (3.2–3.6%) was obtained by adsorption, but the encapsulation efficiency was only 12–13%. Furthermore, this adsorption process is preferable to rule out due to the reduction of the antibacterial activity of STR. This is probably the consequence of the poor protection against environmental conditions (e.g., oxidation processes) and the easy appearance of physicochemical alterations.

The size of the nanoparticles increased when STR was incorporated, but their morphological characteristics remained unchanged (i.e., spherical and rod particles were preferably developed for the ACP and CHAp samples, respectively). The incorporation of STR also led to a remarkable change in the nanoparticles surface charge, which varied from typically negative to positive values. The incorporation of STR also exerted an influence on the decreasing crystallinity and size of the crystalline microdomains. 

A combined release model based on the Higuchi equation from 0–60% of release and a first-order model from 40–100% was appropriate to simulate the experimental STR release curves from the nanoparticles in a PBS medium. The release was faster from ACP particles and in all cases when the drug was incorporated into the outer parts of the nanoparticles (e.g., loaded by solution adsorption or from the reaction mixture). It is important for future applications to consider that the released antibiotic maintains its antibacterial activity when released in the PBS medium.

The nanoparticle extracts in concentrations in the range of 0.1–10 mg/mL did not show cytotoxic effects, which was in agreement with the fact that STR is routinely used as a prophylactic antibiotic for cell culture. Nevertheless, a decrease in cell viability (20–40%) was detected in the highly concentrated extracts. STR-loaded nanoparticles may offer potential for the treatment of cancer cells and control over their viability, through the exploitation of STR’s ability to inhibit protein synthesis in ribosomes and the structural and functional similarities between mitochondria and bacteria. 

Saos-2 cells (derived from osteosarcoma) were able to endocytic nanoparticles loaded with STR. FTIR microspectroscopy analyses allowed the evaluation of the cell damage caused by STR activity inside the cells. Biochemical alterations inside the cells were more noticeable in the loaded ACP nanoparticles.

The new STR-loaded nanoparticles were efficiently incorporated in polymeric matrices such as polylactide electrospun scaffolds. The characteristics of the PLA microfibers (e.g., hydrophobicity and diameter) were influenced when processing was performed in the presence of HAp nanoparticles. Interestingly, STR could be released from the prepared scaffolds with similar behavior (although with a lower rate) to that observed in the nanoparticles. 

In summary, hydroxyapatite nanoparticles medicated with the STR antibiotic appear more efficient when prepared from the precipitation of drug-loaded phosphate solutions. STR maintains its activity and can be efficiently released into a PBS medium. Several biomedical applications can be proposed for these new medicated nanoparticles, such as, for example, in tissue engineering, to provide scaffolds with antibacterial and antitumor properties. 

It should be pointed out that the new scaffolds can provide several advantages with re-spect to related systems: (a) the use of hydroxyapatite as an encapsulating agent, a feature that exploits its natural presence in the human body and the absence of an immunologic response; (b) the easy internalization of hydroxyapatite nanoparticles in cells; (c) the easy and economical production of the proposed scaffolds; and (d) the appropriate protection and relatively low release rate of the encapsulated drug. Furthermore, the results attained with STR corroborated the previous observations performed with chloramphenicol about the expectations of the employment of these antibiotics as anticancer agents [30,31,48,53], exploiting the similarities between mitochondria and bacteria.

## Figures and Tables

**Table 1:** Streptomycin (STR)-loaded in ACP and cHAp nanoparticles using different loading routes during their synthesis. [*] indicates the loading path of the drug in the nanoparticle.

STR Loaded Path [*]	ACP		cHAp	
	EE (%)	DL (%)	EE (%)	DL (%)
(PO_4_)^3−^ dissolution, (ps)	21.5	1.8	25.9	2.2
Ca^2+^ dissolution, (cs)	16.9	1.4	21.8	1.9
Reaction mixture, (rm)	5.4	0.5	7.7	0.7
Adsorption, (as)	11.7	3.2	13.2	3.6

**Table 2:** Degree of crystallinity (*X_C_*) and crystallite size (*L*) of the unloaded and STR-loaded samples. [*] indicates the loading path of the drug in the nanoparticle.

Sample, [*]	*X_C_* (%)		*L* (nm)	
	ACP	cHAp	ACP	cHAp
Without STR	24.0	78.4	11.8	28.3
STR in Ca^2+^ dissolution, (cs)	20.0	76.7	8.6	24.4
STR in (PO_4_)^3−^ dissolution, (ps)	20.0	72.2	7.8	23.8
STR in the reaction mixture, (rm)	6.8	63.2	6.2	19.9
STR by adsorption, (as)	9.5	66.4	6.7	21.5

**Table 3:** Release kinetic parameters for ACP and cHAp nanoparticles loaded with STR. [*] indicates the loading path of the drug in the nanoparticle.

STR Loading, [*]	ACP				cHAp			
	*k_H_* (h^−0.5^)	*r*	*k*_1_ (h^−1^)	*r*	*k_H_* (h^−0.5^)	*r*	*k*_1_ (h^−1^)	*r*
Ca^2+^ dissolution, (cs)	0.239	0.982	0.047	0.991	0.095	0.936	0.040	0.997
(PO_4_)^3−^ dissolution, (ps)	0.261	0.988	0.038	0.979	0.099	0.988	0.028	0.960
Reaction mixture, (rm)	2.000	1.000	0.073	0.951	1.275	0.974	0.062	0.994
Adsorption, (as)	2.123	0.981	0.074	0.963	1.328	0.959	0.039	0.988

**Table 4:** Release kinetic parameters in a PBS medium for PLA electrospun samples incorporating ACP_ps_ and cHAp_ps_ nanoparticles.

Fiber	Release Kinetic Parameters			
	*k_H_* (h^−0.5^)	*r*	*k_1_* (h^−1^)	*r*
PLA/ACP_ps_	0.641	0.999	0.029	0.960
PLA/cHAp_ps_	0.544	0.997	0.020	0.961

## Data Availability

Not applicable.
